# Pericardite Constritiva com Necrose Caseosa: Uma Apresentação Rara e Pouco Conhecida

**DOI:** 10.36660/abc.20250163

**Published:** 2025-11-19

**Authors:** Plínio José Whitaker Wolf, Edileide Barros Correia, Marcos de Oliveira Vasconcellos, Larissa Ventura Ribeiro Bruscky, Ana Cristina de Souza Murta, Yoná Afonso Francisco, Eduardo Mikio Sassaki, Sara Regina Alcalde Domingos, Natan Alevato Donadon, Gerson Miranda, Raphael Rossi, Victor Bemfica de Mello Mattos, Fernanda de Brito Fortuna

**Affiliations:** 1 Instituto Dante Pazzanese de Cardiologia São Paulo SP Brasil Instituto Dante Pazzanese de Cardiologia, São Paulo, SP – Brasil; 2 Centro de Referência e Treinamento DST/Aids-SP São Paulo SP Brasil Centro de Referência e Treinamento DST/Aids-SP, São Paulo, SP – Brasil

**Keywords:** Pericardite Constritiva, Pericárdio, Insuficiência Cardíaca

## Abstract

Dá-se o nome de necrose caseosa da válvula mitral à ocorrência de liquefação do cálcio presente em seu anel fibroso e consequente formação de um pseudotumor com conteúdo pastoso e espesso em seu interior. Apesar de raro, tal processo degenerativo já é amplamente descrito no contexto da valva mitral, contudo, é pouco conhecido na condição da pericardite constritiva. Descrevemos uma série de sete casos que apresentaram quadro de pericardite constritiva associada à necrose caseosa. Dos sete pacientes, seis eram masculinos, com idade média de 42±14 anos, todos em classe funcional III/IV, com clínica relacionada à síndrome restritiva. A tomografia de tórax evidenciou intensa calcificação pericárdica com necrose caseosa, confirmada pela pericardiectomia.

## Graphical abstract

**Figura Central:**
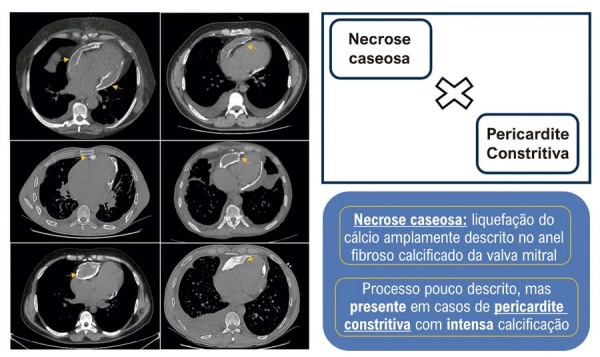
Pericardite Constritiva com Necrose Caseosa: Uma Apresentação Rara e Pouco Conhecida

Tomografias comp. de tórax demonstrando pericardite constritiva calcificada com necrose caseosa. Setas amarelas destacando áreas com necrose caseosa.

## Introdução

A calcificação do anel mitral (CAM) é definida como uma degeneração crônica do anel fibroso da valva,^1^ sendo a necrose caseosa (NC) do anel mitral (NCVM), por sua vez, uma rara variante desta condição, em que ocorre liquefação do conteúdo calcificado dessa estrutura.^2^ A NC, já amplamente descrita na valva mitral, é rara e pouco reconhecida no contexto da pericardite constritiva (PC) calcificada.

Descrevemos uma série de casos evidenciando essa incomum associação entre a PC e a NC.

## Relato de Casos

Descrevemos sete pacientes portadores de PC associada à NC, de maneira observacional e retrospectiva, em centro único, referência em cardiomiopatias no Brasil. Dentre eles, seis eram masculinos, com idade média de 42±14 anos. A presença de comorbidades foi infrequente, verificada em quatro deles, sendo as mais comuns, o hipotireoidismo e o tabagismo ( Tabela 1 ).

**Tabela 1:** – Características basais clínicas, ecocardiográficas, laboratoriais e anatomopatológicas

Paciente	Sexo/Idade	AP	CF (NYHA) Pré Cx	Início queixa (meses)	ECOTT Pré Cx	FEVE Após Cx	FA	Biópsia Pericárdica	PCR (mg/dL)	NT-pro BNP (pg/mL)	CF (NYHA) Após Cx
P1	M 62 anos	HipoT, Obesidade, HAS, DRC	III	2	FEVE: 66%; AE: 50 mL/m ^2^ ; PSAP: ausente; AR+/SB+	59%	Sim	Pericardite crônica, fibrose e calcificação. Sem malignidade ou granuloma	2,0	2350	II
P2	M 35 anos	Ausente	III	2	FEVE: 58%; AE: 26 mL/m ^2^ ; PSAP: ausente; AR+/SB+	61%	Não	Pericardite crônica, fibrose e calcificação. Sem malignidade ou granuloma	1,9	115	I
P3	M 38 anos	Tabagismo	IV	24	FEVE: 58%; AE: 61 mL/m ^2^ ; PSAP: 39 mmHg; Disfunção VD; AR+/SB+	60%	Não	Pericardite crônica, fibrose e calcificação. Sem malignidade ou granuloma	0,5	1329	III
P4	F 60 anos	HipoT, DLP, HAS	III	24	FEVE: 62%; AE: 50 mL/m ^2^ ; PSAP: 35 mmHg; AR+/SB+	62%	Não	Pericardite crônica, fibrose e calcificação. Sem malignidade ou granuloma	0,5	1500	I
P5	M 40 anos	Ausente	III	12	FEVE: 46%; AE: 43 mL/m ^2^ ; PSAP: 34 mmHg; Disfunção VD; AR+/SB+	47%	Não	Pericardite crônica, fibrose e calcificação. Sem malignidade ou granuloma	0,9	135	II
P6	M 39 anos	HipoT, DRC	III	24	FEVE: 24%; AE: 65 mL/m ^2^ ; PSAP: 40 mmHg; Disfunção VD; AR+/SB+	50%	Sim	Pericardite crônica, fibrose e calcificação, degeneração mixoide. Sem malignidade ou granuloma	0,5	1650	I
P7	M 22 anos	Ausente	II	6	FEVE: 65%; AE: 46 mL/m ^2^ ; PSAP: NA; AR+/SB+	65%	Não	Pericardite crônica, fibrose e calcificação. Sem malignidade ou granuloma	1,8	NA	I

*>3 meses da cirurgia. AP: antecedentes pessoais; AR: annulus reverso; AE: volume átrio esquerdo; CF: classe funcional; Cx: cirurgia; DLP: dislipidemia; DRC: doença renal crônica; ECOTT: ecocardiograma transtorácico; FA: fibrilação atrial; FEVE: fração de ejeção de ventrículo esquerdo; HAS: hipertensão arterial sistêmica; HipoT: hipotireoidismo; IC: insuficiência cardíaca; PCR: proteína C reativa; PSAP: pressão sistólica artéria pulmonar; NA: não avaliado; NT-pro BNP: porção N-terminal proBNP; NYHA: New York Heart Association; SB: septal bounce; VD: ventrículo direito.

Síndrome restritiva foi a manifestação inicial de forma unânime, expressa por dispneia progressiva, classe funcional III/IV da NYHA, predominando os sinais de insuficiência cardíaca (IC) direita, estando presente, na totalidade dos casos, edema de membros inferiores, ascite, derrame pleural, hepatomegalia, turgência jugular e refluxo hepatojugular. Não houve descrição de sinal de Kussmaul ou pulso paradoxal em nenhum dos pacientes. O tempo médio entre o início dos sintomas e a admissão hospitalar foi de pelo menos 2 meses (média de 13,4±10,4 meses), denotando quadro clínico arrastado e progressivo.

Não foram observadas alterações importantes nos exames laboratoriais, incluindo a contagem de leucócitos e proteína C reativa (PCR) (1,15±0,7 mg/dL). O NT-proBNP médio foi de 1179±887 pg/mL.

O ecocardiograma transtorácico (ECOTT), confirmou o diagnóstico de PC, com os achados típicos de pericárdio espessado, calcificado, além de sinais de interdependência ventricular. A fração de ejeção de ventrículo esquerdo (FEVE) se encontrava reduzida (inferior a 40%) em apenas um paciente, com média de 54±14,8%, entretanto, em 42% dos casos, havia disfunção do ventrículo direito (VD). Outros achados foram aumento de átrio esquerdo (85%), com volume médio de 48±12 mL/m ^2^ e hipertensão pulmonar (57%). Foram visualizadas imagens hiperecogênicas heterogêneas, aderidas ao pericárdio, gerando restrição das câmaras, sobretudo o VD.

**Figura 1:**
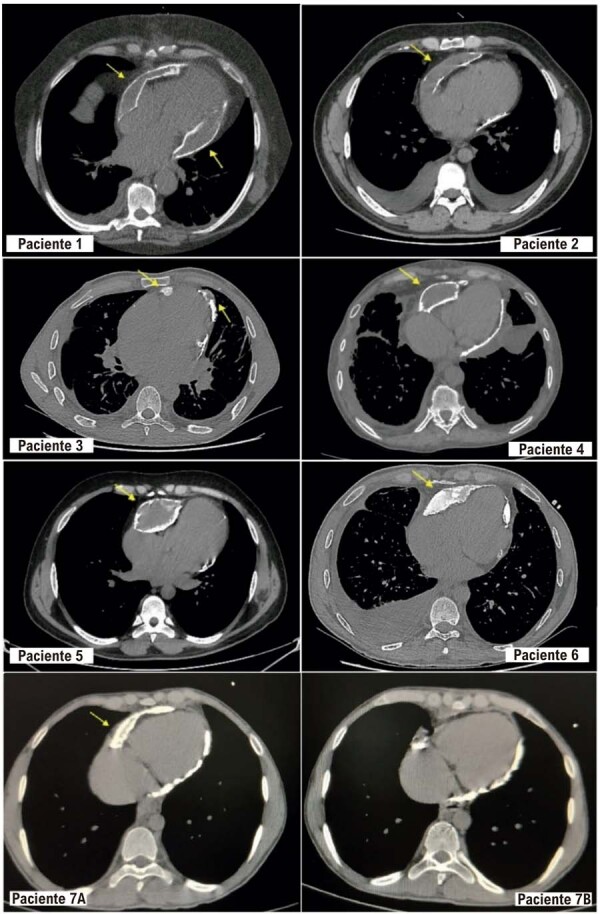
– Tomografias comp. de tórax demonstrando pericardite constritiva calcificada com necrose caseosa. Tomografias de tórax evidenciando pericárdio espessado, calcificado, associado a “pseudotumores” com contornos irregulares de conteúdo heterogêneo hipodenso (setas amarelas). “Paciente 7A” e “Paciente 7B” se referem ao mesmo paciente, antes e após a pericardiectomia, respectivamente.

A radiografia de tórax demonstrava radiopacidade contornando a silhueta cardíaca. A tomografia computadorizada de tórax (TC) ( Figura 1 ) confirmou espessamento pericárdico com hiperdensidade importante, associado a “pseudotumores” com contornos irregulares e conteúdo heterogêneo mais hipodenso em seu interior, causando abaulamento da silhueta cardíaca.

No intraoperatório da pericardiectomia, realizada em todos, foi constatado pericárdio espessado, rígido, calcificado e aderido às estruturas adjacentes. Observou-se exposição de substância esbranquiçada, pastosa, semelhante a “pasta-de-dente”, em moderada-grande quantidade, surgida dos “pseudotumores” presentes no pericárdio, a chamada NC ou o “caseum”. Evidenciou-se recuperação da função ventricular após o procedimento.

No pós-operatório tardio, todos apresentaram melhora significativa dos sintomas, evoluindo, em sua maioria, para classe funcional NYHA I-II, principalmente a partir dos 3 meses da cirurgia. A FEVE reduzida, observada em um único paciente, normalizou-se após 24 horas da cirurgia (24% para 50%). Todos se mantiveram oligossintomáticos no seguimento médio de 2,7 anos.

Os pacientes foram avaliados quanto à etiologia da pericardite, incluindo tuberculose (TB), por meio da análise anátomo patológica do pericárdio, onde não foram evidenciados, em nenhum dos pacientes, granulomas ou malignidade. Baciloscopia e cultura para micobactéria negativas nos materiais, além de ausência de manifestações clínicas, prévias ou posteriores (febre, tosse, perda de peso e sudorese noturna) que sugerissem doenças infecciosas em atividade, como a TB.

## Discussão

Descrevemos sete casos da rara e pouco reconhecida associação da NC com a PC, cuja descrição é escassa na literatura, reportada em apenas uma publicação.^3^

No entanto, a descrição desse processo degenerativo já é definitivamente consolidada no que diz respeito ao anel mitral.^1 , 2 , 4 , 5^ A CAM ocorre em 8 a 15% da população geral,^6^ enquanto que a NCVM, mais rara, tem uma prevalência ainda desconhecida e subestimada, ocorrendo em torno 0,06-0,07%,^1 , 4^ e é mais prevalente em mulheres, idosos, hipertensos, doentes renais crônicos e naqueles com alterações no metabolismo do cálcio.^1 , 2^ Geralmente é assintomática e a multimodalidade de imagem é fundamental para o diagnóstico.^2^

Neste estudo, diferindo da NC confinada à valva mitral já amplamente reconhecida, descrevemos a ocorrência da mesma na PC calcificada. A PC é caracterizada por uma inflamação do pericárdio que, na maioria das vezes, leva à fibrose, com ou sem calcificação, perdendo sua elasticidade, levando à disfunção diastólica ventricular.^7 - 9^ De fato, pode ocorrer após qualquer processo patológico pericárdico, cujo risco de progressão para PC é dependente da etiologia: baixo (<1%) na pericardite viral/idiopática, intermediário (2-5%) nas causas neoplásicas/autoimunes e alto (20-30%) em etiologias bacterianas. Registros de países desenvolvidos demonstram que as principais causas incluem as cirurgias cardíacas (11-37%), radioterapia (9-31%) e etiologias viral ou idiopática (42-49%). A tuberculose, embora causa rara de pericardite nesses países (<4%), ainda é considerada uma clássica etiologia em regiões menos desenvolvidas (50-70%), evoluindo, mesmo com tratamento adequado, à PC em 17 a 40% dos casos.^8 - 12^ Independentemente da etiologia, o espessamento e a perda da elasticidade do pericárdio levam a um estado de interdependência ventricular, bem como a uma redução de complacência pericárdica, com consequente redução do enchimento das câmaras cardíacas e do retorno venoso (refletindo em sinais e sintomas de síndrome restritiva, sobretudo a direita, conforme apresentaram os pacientes relatados).^8 , 10 - 12^ Os sinais e sintomas de IC direita podem ser encontrados em outros diagnósticos diferenciais, como a cardiomiopatia restritiva,^8^ mas os evidentes achados ecocardiográficos dos sete pacientes relacionados à interdependência ventricular, *septal bounce* e *annulus* reverso, associados à doença pericárdica evidente, trouxeram a confirmação diagnóstica de PC.

Na PC, o pericárdio se encontra espessado em 80% dos casos^9^ e calcificado em 11 a 70% das situações, sobretudo em casos de PC idiopática, relacionada à radiação ou TB.^12 , 13^ Na casuística, o pericárdio se encontrava espessado e calcificado na totalidade dos pacientes. Além disso, foi observada a presença de estruturas compatíveis com “pseudotumores” no pericárdio nos exames de imagem, cujo conteúdo era composto pelo “caseum”, confirmado na cirurgia, configurando a NC.

O mecanismo fisiopatológico da NC não é completamente compreendido. A hipótese levantada na NCVM lastreia-se na liquefação do material calcificado do anel mitral. A hipercolesterolemia e macrófagos carregados de lipídeos poderiam levar à formação de uma cavidade com material pastoso, semelhante à aparência de “pasta-de-dente”, composta por ácidos graxos, colesterol e cálcio.^1 , 4 , 14^ Acreditamos que, nos casos de PC calcificada, mecanismo fisiopatológico semelhante esteja envolvido, pela intensa calcificação compartilhada nesses cenários.

O caráter crônico da condição pode ser inferido pelo longo tempo dos sintomas associado a baixa inflamação sistêmica, refletido pelas reduzidas dosagens de PCR.

Os níveis de peptídeos natriuréticos não foram correlacionados à gravidade dos sintomas, provavelmente pela restrição da dilatação das câmaras cardíacas pela pericardite, como já descrito previamente em outras publicações no contexto da PC.^10 , 12^ Observou-se melhora clínica muito expressiva após a pericardiectomia, permanecendo 85% dos pacientes em NYHA≤II, associado a ausência de óbitos ocorridos no periprocedimento (a literatura relata mortalidade em torno de 6-12%^10^), o que reafirma a crescente indicação (incremento na realização de pericardiectomia de 2% para 34% na PC) e o benefício desse procedimento, mesmo diante de intensa calcificação e NC.^12^

Em relação à etiologia, esta foi considerada idiopática em todos os casos, sendo excluídas tuberculose e malignidade, além de outras causas tratáveis, por meio da avaliação laboratorial, do histórico clínico e pela análise do material da biópsia, que confirmou calcificação, fibrose e inflamação inespecífica. Tais achados corroboram com a possibilidade de que a PC possa ocorrer em consequência de qualquer agressão pericárdica^9^ e reflete outras séries de casos PC calcificada, onde a causa idiopática foi a mais prevalente.^15^ Ressalta-se que, apesar da TB ser uma importante causa de PC em países subdesenvolvidos (sendo, portanto, essencial seu rastreio), o achado de NC na PC não tem relação direta com essa etiologia e nem mesmo com seus típicos granulomas caseosos. Embora haja similaridade na nomenclatura, a NC em questão é secundária à liquefação do material calcificado, não sendo um processo exclusivo da atividade da TB.^1^

Concluímos que a ocorrência de NC pode estar presente nos casos de intensa calcificação pericárdica e que sua ocorrência não implica em mudança de estratégia terapêutica e prognóstico. O entendimento aprofundado desta condição demanda mais estudos, entretanto, o pioneirismo dessa série de casos pode contribuir para posteriores discussões e pesquisas.
