# Supplementary material for: Pericardite Constritiva com Necrose Caseosa: Uma Apresentação Rara e Pouco Conhecida
Source: Arq Bras Cardiol. 2025 Nov 19;122(10):e20250163. [Article in Portuguese] doi: 10.36660/abc.20250163 (PMC12677734; doi:10.36660/abc.20250163)
Supplement: Translation

# Translation: Constrictive Pericarditis with Caseous Necrosis: A Rare and Underrecognized Presentation

**DOI:** 10.36660/abc.20250163i

**Authors:** Plínio José Whitaker Wolf, Edileide Barros Correia, Marcos de Oliveira Vasconcellos, Larissa Ventura Ribeiro Bruscky, Ana Cristina de Souza Murta, Yoná Afonso Francisco, Eduardo Mikio Sassaki, Sara Regina Alcalde Domingos, Natan Alevato Donadon, Gerson Miranda, Raphael Rossi, Victor Bemfica de Mello Mattos, Fernanda de Brito Fortuna

**Affiliations:** 1 Brazil Instituto Dante Pazzanese de Cardiologia, São Paulo, SP – Brazil; 2 Brazil Centro de Referência e Treinamento DST/Aids-SP, São Paulo, SP – Brazil

**Keywords:** Constrictive Pericarditis, Pericardium, Heart Failure

## Abstract

Caseous necrosis of the mitral valve is the term used to describe the liquefaction of calcium present in its fibrous ring and the consequent formation of a pseudotumor with a thick, pasty content within. Although rare, this degenerative process has been widely described in the context of the mitral valve; however, it is little known in the context of constrictive pericarditis. We describe a series of seven cases that presented with constrictive pericarditis associated with caseous necrosis. Of the seven patients, six were male, with a mean age of 42±14 years, all in functional class III/IV, with clinical signs related to restrictive syndrome. Chest tomography showed intense pericardial calcification with caseous necrosis, confirmed by pericardiectomy.

## Introduction

Mitral annular calcification (MAC) is defined as a chronic degeneration of the fibrous annulus of the valve,^1^with caseous necrosis of the mitral annulus (CNVM), in turn, being a rare variant of this condition, in which liquefaction of the calcified content of this structure occurs.^2^Caseous necrosis, already widely described in the mitral valve, is rare and little recognized in the context of calcific constrictive pericarditis.

We describe a series of cases demonstrating this unusual association between constrictive pericarditis (CP) and caseous necrosis (CN).

## Case Report

We describe seven patients with CP associated with CN, in an observational and retrospective manner, in a single center, a reference center for cardiomyopathies in Brazil. Among them, six were male, with a mean age of 42±14 years. The presence of comorbidities was uncommon, observed in four of them, the most common being hypothyroidism and smoking ( Table 1 ).

**Table 1:** – Baseline clinical, echocardiographic, laboratory, and anatomopathological characteristics

Patient	Sex/Age	PH	FC (NYHA) Pre Cx	Onset of complaint (months)	TTE Pre Cx	LVEF After Cx	AF	Pericardial Biopsy	CRP (mg/dL)	NT-pro BNP (pg/mL)	FC (NYHA) After Cx
P1	M 62 years old	Hypotonic, Obesity, Hypertension, CKD	III	2	LVEF: 66%; LA: 50 mL/m ^2^ ; PSAP: absent; AR+/SB+	59%	Yes	Chronic pericarditis, fibrosis, and calcification. No malignancy or granuloma.	2.0	2350	II
P2	M 35 years old	Absent	III	2	LVEF: 58%; LA: 26 mL/m ^2^ ; PSAP: absent; AR+/SB+	61%	No	Chronic pericarditis, fibrosis, and calcification. No malignancy or granuloma.	1.9	115	I
P3	M 38 years old	Smoking	IV	24	LVEF: 58%; LA: 61 mL/m ^2^ ; PSAP: 39 mmHg; RV dysfunction; AR+/SB+	60%	No	Chronic pericarditis, fibrosis, and calcification. No malignancy or granuloma.	0.5	1329	III
P4	F 60 years old	Hypotonic, DLP, SAH	III	24	LVEF: 62%; LA: 50 mL/m ^2^ ; PSAP: 35 mmHg; AR+/SB+	62%	No	Chronic pericarditis, fibrosis, and calcification. No malignancy or granuloma.	0.5	1500	I
P5	M 40 years old	Absent	III	12	LVEF: 46%; LA: 43 mL/m ^2^ ; PSAP: 34 mmHg; RV dysfunction; AR+/SB+	47%	No	Chronic pericarditis, fibrosis, and calcification. No malignancy or granuloma	0.9	135	II
P6	M 39 years old	Hypotonic, CKD	III	24	LVEF: 24%; LA: 65 mL/m ^2^ ; PSAP: 40 mmHg; RV dysfunction; AR+/SB+	50%	Yes	Chronic pericarditis, fibrosis and calcification, myxoid degeneration. No malignancy or granuloma.	0.5	1650	I
P7	M 22 years old	Absent	II	6	LVEF: 65%; LA: 46 mL/m ^2^ ; PSAP: NA; AR+/SB+	65%	No	Chronic pericarditis, fibrosis, and calcification. No malignancy or granuloma.	1.8	IN	I

*>3 months after surgery. PH: personal history; AR: annulus reversion; LA: left atrial volume; FC: functional class; Cx: surgery; DLP: dyslipidemia; CKD: chronic kidney disease; TTE: transthoracic echocardiogram; AF: atrial fibrillation; LVEF: left ventricular ejection fraction; SAH: systemic arterial hypertension; HypoT: hypothyroidism; HF: heart failure; CRP: C-reactive protein; PASP: pulmonary artery systolic pressure; NA: not assessed; NT-pro BNP: N-terminal pro BNP; NYHA: New York Heart Association; SB: septal bounce; RV: right ventricle.

Restrictive syndrome was the unanimous initial manifestation, expressed by progressive dyspnea, NYHA functional class III/IV, with predominant signs of right-sided heart failure (HF). Lower limb edema, ascites, pleural effusion, hepatomegaly, jugular distension, and hepatojugular reflux were present in all cases. No Kussmaul sign or pulsus paradoxus was reported in any of the patients. The mean time between symptom onset and hospital admission was at least 2 months (mean 13.4±10.4 months), indicating a prolonged and progressive clinical picture.

No significant changes were observed in laboratory tests, including leukocyte count and C-reactive protein (CRP) (1.15±0.7 mg/dL). The mean NT-proBNP was 1179±887 pg/mL.

The transthoracic echocardiogram (TTE) confirmed the diagnosis of constrictive pericarditis, with typical findings of thickened, calcified pericardium, in addition to signs of ventricular interdependence. The left ventricular ejection fraction (LVEF) was reduced (less than 40%) in only one patient, with a mean of 54 ± 14.8%; however, in 42% of cases, there was right ventricle (RV) dysfunction. Other findings were left atrial enlargement (85%), with a mean volume of 48 ± 12 mL/m ^2^ and pulmonary hypertension (57%). Heterogeneous hyperechoic images were observed, adhering to the pericardium, causing restriction of the chambers, especially the RV.

A chest radiograph demonstrated radiopacity surrounding the cardiac silhouette. A chest computed tomography (CT) scan ( Figure 1 ) confirmed pericardial thickening with significant hyperdensity, associated with “pseudotumors” with irregular contours and heterogeneous, more hypodense contents within them, causing bulging of the cardiac silhouette.

**Figure 1:** – Chest CT scans demonstrating calcific constrictive pericarditis with caseous necrosis. Chest CT scans showing thickened, calcified pericardium associated with irregularly contoured pseudotumors containing heterogeneous, hypodense contents (yellow arrows). “Patient 7A” and “Patient 7B” refer to the same patient, before and after pericardiectomy, respectively.

During pericardiectomy, performed in all patients, the pericardium was found to be thickened, rigid, calcified, and adherent to adjacent structures. A moderate to large amount of a whitish, pasty substance resembling toothpaste was observed, originating from the “pseudotumors” present in the pericardium, known as CN or “caseum.” Recovery of ventricular function was evident after the procedure.

In the late postoperative period, all patients showed significant improvement in symptoms, most progressing to NYHA functional class I-II, mainly after 3 months of surgery. The reduced LVEF, observed in a single patient, normalized 24 hours after surgery (24% to 50%). All patients remained mildly symptomatic during the mean follow-up of 2.7 years.

Patients were evaluated for the etiology of pericarditis, including tuberculosis (TB), through anatomical pathology of the pericardium, which revealed no granulomas or malignancy in any of the patients. Bacilloscopy and culture for mycobacteria were negative in the samples, and there were no previous or subsequent clinical manifestations (fever, cough, weight loss, and night sweats) suggestive of active infectious diseases, such as TB.

## Discussion

We describe seven cases of the rare and little recognized association of CN with constrictive pericarditis, whose description is scarce in the literature, reported in only one publication.^3^

However, the description of this degenerative process is already definitively consolidated concerning the mitral annulus.^1 , 2 , 4 , 5^MAC occurs in 8 to 15% of the general population,^6^while CNVM, which is rarer, has a prevalence that is still unknown and underestimated, occurring at around 0.06-0.07%,^1^and is more prevalent in women, the elderly, hypertensive patients, chronic kidney patients, and those with alterations in calcium metabolism.^1 , 2^It is usually asymptomatic, and multimodality imaging is essential for diagnosis.^2^

In this study, unlike the already widely recognized CN confined to the mitral valve, we describe its occurrence in calcific constrictive pericarditis. CP is characterized by inflammation of the pericardium that, in most cases, leads to fibrosis, with or without calcification, loss of elasticity, and ventricular diastolic dysfunction.^7 - 9^In fact, it can occur after any pericardial pathological process, the risk of progression to CP depending on the etiology: low (<1%) in viral/idiopathic pericarditis, intermediate (2-5%) in neoplastic/autoimmune causes, and high (20-30%) in bacterial etiologies. Records from developed countries show that the main causes include cardiac surgery (11-37%), radiotherapy (9-31%), and viral or idiopathic etiologies (42-49%). TB, although a rare cause of pericarditis in these countries (<4%), is still considered a classic etiology in less developed regions (50-70%), progressing, even with appropriate treatment, to CP in 17-40% of cases.^8 - 12^Regardless of the etiology, thickening and loss of elasticity of the pericardium lead to a state of ventricular interdependence, as well as a reduction in pericardial compliance, with a consequent reduction in the filling of the cardiac chambers and venous return (reflecting in signs and symptoms of restrictive syndrome, especially on the right, as presented by the reported patients).^8 , 10 - 12^Signs and symptoms of right-sided HF may be found in other differential diagnoses, such as restrictive cardiomyopathy,^8^but the evident echocardiographic findings of the seven patients related to ventricular interdependence, septal bounce, and annulus reversion, associated with evident pericardial disease, brought the diagnostic confirmation of constrictive pericarditis.

In CP, the pericardium is thickened in 80% of cases^9^and calcified in 11 to 70% of cases, especially in cases of idiopathic CP, related to radiation or TB.^12 , 13^In the case series, the pericardium was thickened and calcified in all patients. Furthermore, the presence of structures consistent with “pseudotumors” in the pericardium was observed on imaging exams, the contents of which consisted of “caseum,” confirmed surgically, constituting CN.

The pathophysiological mechanism of CN is not fully understood. The hypothesis raised in CNVM is based on the liquefaction of calcified material in the mitral annulus. Hypercholesterolemia and lipid-laden macrophages could lead to the formation of a cavity with a pasty material, similar to the appearance of “toothpaste,” composed of fatty acids, cholesterol, and calcium.^1 , 4 , 14^We believe that, in cases of calcified CP, a similar pathophysiological mechanism is involved, due to the intense calcification shared in these scenarios.

The chronic nature of the condition can be inferred by the long duration of symptoms associated with low systemic inflammation, reflected by reduced CRP levels.

Natriuretic peptide levels were not correlated with symptom severity, probably due to the restriction of dilation of the cardiac chambers by pericarditis, as previously described in other publications in the context of CP.^10 , 12^A very significant clinical improvement was observed after pericardiectomy, with 85% of patients remaining in NYHA≤II, associated with the absence of deaths occurring in the periprocedure (literature reports mortality around 6-12%^10^), which reaffirms the growing indication (increase in the performance of pericardiectomy from 2% to 34% in CP) and the benefit of this procedure, even in the face of intense calcification and CN.^12^

Regarding the etiology, it was considered idiopathic in all cases, with TB and malignancy, as well as other treatable causes, being excluded through laboratory evaluation, clinical history, and analysis of the biopsy material, which confirmed calcification, fibrosis, and nonspecific inflammation. These findings corroborate the possibility that CP can occur as a consequence of any pericardial injury.^9^and reflects other case series of calcified CP, where the idiopathic cause was the most prevalent.^15^It should be noted that, although TB is an important cause of CP in underdeveloped countries (and therefore essential for screening), the finding of CN in CP is not directly related to this etiology or even to its typical caseous granulomas. Although there is a similarity in nomenclature, the CN in question is secondary to the liquefaction of calcified material and is not a process exclusive to TB activity.^1^

We conclude that CN may occur in cases of intense pericardial calcification and that its occurrence does not imply a change in therapeutic strategy or prognosis. A deeper understanding of this condition requires further studies; however, the pioneering nature of this case series can contribute to further discussion and research.
